# MicroRNA-495 suppresses pre-eclampsia via activation of p53/PUMA axis

**DOI:** 10.1038/s41420-022-00874-0

**Published:** 2022-03-25

**Authors:** Yi Zhao, Ge Zhao, Weiwei Li

**Affiliations:** Department of Obstetrics, the First Hospital of China Medical University, Shenyang, 110001 P. R. China

**Keywords:** Molecular biology, Medical research

## Abstract

Linkage between microRNAs (miRNAs) and pre-eclampsia (PE) has been documented. Here, we focused on miR-495 in PE and its underlying mechanism in regulation of trophoblast cells. Expression of miR-495, HDAC2, p53 and PUMA was determined in collected placental tissue samples. Loss- and gain-function was performed to determine the roles of miR-495, HDAC2, p53, and PUMA in biological processes of HTR8/SVneo cells and primary trophoblast cells. The relationships among miR-495, HDAC2, and p53 were pinpointed. PE patients presented with higher expression of miR-495, p53, and PUMA in placental tissues, but lower HDAC2. miR-495 negatively targeted HDAC2 expression. HDAC2 suppressed p53 expression *via* deacetylation. Overexpression of miR-495, p53, or PUMA inhibited biological properties of HTR8/SVneo cells and primary trophoblast cells, while opposite trends were observed in response to oe-HDAC2. In conclusion, miR-495 knockdown can suppress p53/PUMA axis by targeting HDAC2 to enhance biological behaviors of trophoblast cells, which may prevent occurrence of PE.

## Introduction

As a disorder occurred during pregnancy, pre-eclampsia (PE) serves a major cause of maternal mortality and morbidity [1]. New-onset high blood pressure and proteinuria, features of PE, often take place at more than 20 weeks of pregnancy [2]. It is reported that PE exerts effects on 2–7% of all pregnant women worldwide [3]. PE is a pregnancy-related complication and featured by imbalance in angiogenesis and systemic inflammation [4]. The aberrant trophoblast homeostasis bears great responsibility for PE development [5]. PE is associated with deficient placentation that is attributed to the inability of cytotrophoblast to obtain offensive phenotype and to reconstruct the uterine spiral arteries [6]. Current treatments for PE such as hypertension relief, epilepsy prevention, and aspirin usage might only modestly reduce the risk of PE [7]. Insufficiency in trophoblast invasion could promote the progression of PE. Understanding the underlying mechanism of trophoblast proliferation and invasion is therefore critical to the possible treatment for PE.

MicroRNAs (miRNAs) have been reported to make great contributions to PE development as well as exert crucial action in managing biological functions of trophoblast cells [8–10]. The interactions between miRNA and target genes engaged in the pathogenesis of PE have been reviewed previously [11]. For example, the expression of miR-23a shows a significant upregulation in placental tissues of PE patients [12]. It is of interest that miR-495 is revealed to be highly expressed in exosomes derived from peripheral blood of PE patients [13]. Bioinformatics prediction showed a targeting association between miR-495 and histone deacetylase 2 (HDAC2). HDACs play critical roles in remodeling chromatin and regulating gene- and cellular-signaling pathways, in which HDAC2 is important for learning and memory abilities during late gestation [14, 15]. Notably, a decline in HDAC2 expression is seen in placental tissues of severe PE patients [16]. HDAC2 participates in the modulation of placental P-glycoprotein both in vitro and in vivo [17]. In addition, previous study reported that HDAC2 protein interacted with p53 in the human spermatogenesis [18]. The p53-mediated trophoblast apoptosis was reported to be involved in the etiology of PE [19]. Expression of p53-upregulated modulator of apoptosis (PUMA) is also increased in tissues of PE patients [20]. Here, we aimed to investigate the mechanism of how miR-495/HDAC2/p53/PUMA axis functions in the pathogenesis of PE.

## Results

### Higher miR-495 is involved in PE progression

**Fig. 1:**
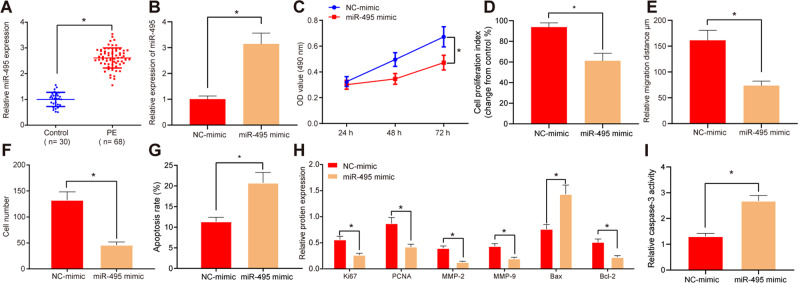
miR-495 expression is increased in PE. **A** Expression of miR-495 in umbilical cord tissues analyzed by RT-qPCR (control *n* = 30, PE *n* = 68). **B** Expression of miR-495 in HTR8/SVneo cells 48 h after transfection via RT-qPCR. **C** HTR8/SVneo cell viability at 24^th^, 48^th^, and 72^nd^ hour after transfection evaluated by MTT assay. **D** HTR8/SVneo cell proliferation 48 h after transfection evaluated by BrdU assay. **E** Migrating ability of HTR8/SVneo cells 24 h after transfection examined using scratch assay. **F** HTR8/SVneo cell invasion 24 h after transfection assessed using transwell assay (200×). **G** HTR8/SVneo cell apoptosis in each group 48 h after transfection analyzed by flow cytometry. **H** Protein expression of proliferation-related factors (Ki67 and PCNA), migration- and invasion-related factors (MMP-2 and MMP-9), and apoptotic factors (Bax and Bcl-2) in HTR8/SVneo cells 48 h after transfection studied by western blot. **I** Caspase-3 activity in HTR8/SVneo cells 48 h after transfection. *p < 0.05 vs. control or NC mimic. The quantitative data were presented as mean ± standard deviation. Difference between two groups was compared using paired *t-*test. Cell experiments were repeated 3 times.

We first focused on the expression of miR-495 in PE. Utilizing RT-qPCR, we discovered elevated miR-495 in umbilical cord tissues and primary trophoblast cells of PE patients than that in the healthy pregnant women (Fig. 1A; Supplementary Fig. S1A).

Next, it was shown that miR-495 expression was upregulated in HTR8/SVneo cells transfected with miR-495 mimic compared with the control, suggesting the cell transfection was successful (Fig. 1B; Supplementary Fig. S2A). 3-(4,5-Dimethyl-2-thiazyl)-2,5-diphenyl-2H-tetrazolium bromide (MTT) assay (Fig. 1C; Supplementary Fig. S2B), bromodeoxyuridine (BrdU) assay (Fig. 1D; Supplementary Fig. S2C), scratch assay (Fig. 1E; Supplementary Figs. S3A and S2D), transwell assay (Fig. 1F; Supplementary Figs. S3B and S2E), and flow-cytometry (Fig. 1G; Supplementary Figs. S3C and S2F) results further demonstrated that cell viability, proliferation, migration, and invasion of HTR8/SVneo cells and primary trophoblast cells were limited in cells transfected with miR-495 mimic, but cell apoptosis was increased.

Further expression determination with Western blot demonstrated that protein levels of Ki67, PCNA, MMP-2, MMP-9, and Bcl-2 were decreased, whereas Bax expression was increased in miR-495 mimic transfected HTR8/SVneo cells and primary trophoblast cells (Fig. 1H; Supplementary Fig. S2G). Meanwhile, the activity of caspase 3 was increased in response to miR-495 mimic transfection (Fig. 1I; Supplementary Fig. S2H). Thus, upregulation of miR-495 limited HTR8/SVneo cell and primary trophoblast-cell proliferation.

### HDAC2 is targeted by miR-495

**Fig. 2:**
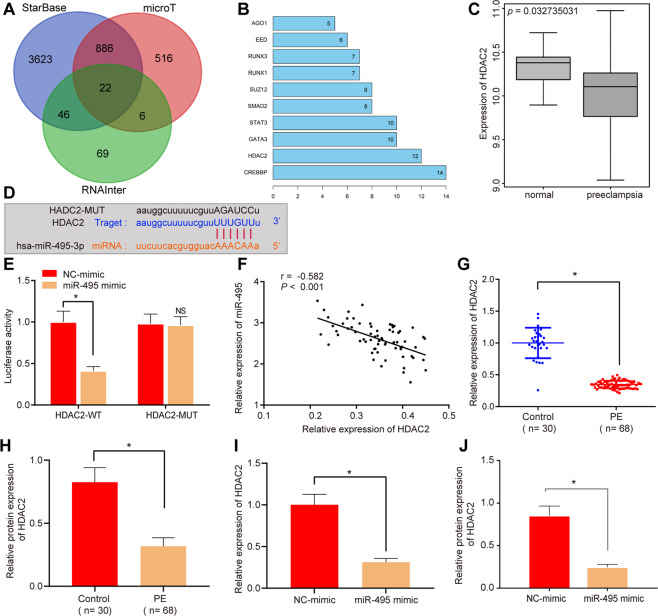
miR-495 negatively regulates HDAC2 expression in HTR8/SVneo cells. **A** StarBase, RNAInter, and microT website prediction on miR-495 target genes displayed in the Venn diagram. **B** STRING to analyze the protein–protein interaction network. X axis represents the degree of interaction of target genes, Y axis shows target genes. **C** HDAC2 expression in microarray GSE48424. X axis represents the sample types; Y axis represents the expression of HDAC2. **D** Binding site between HDAC2 and miR-495 predicted by Starbase. **E** Dual-luciferase reporter gene-assay verification on the relationship between HDAC2 and miR-495. **F** Pearson correlation analysis of HDAC2 and miR-495 through Pearson’s correlation coefficient. **G** RT-qPCR detection on HDAC2 expression in umbilical cord tissues of different groups (control *n* = 30, PE *n* = 68). **H** Western blot analysis on HDAC2 expression in umbilical cord tissues of different groups (control *n* = 30, PE *n* = 68). **I** RT-qPCR detection on HDAC2 expression in HTR8/SVneo cells 48 h after transfection. **J** Western blot analysis on HDAC2 expression in HTR8/SVneo cells 48 h after transfection. *p < 0.05 vs. control or NC mimic. The quantitative data were presented as mean ± standard deviation. Difference between two groups was analyzed by paired *t-*test. Cell experiments were repeated 3 times.

To further evaluate the role of miR-495 in PE, we adopted online websites StarBase, RNAInter, and microT databases to predict the target genes of miR-495, which showed that 22 genes may be bound to miR-495 (Fig. 2A). Protein–protein interaction-network analysis was performed through STRING, which demonstrated that CREBBP and HDAC2 functioned as the hub (Fig. 2B). Differential analysis was conducted on microarray GSE48424, which revealed that HDAC2 was downregulated in PE (Fig. 2C). The StarBase website prediction data indicated binding sites between miR-495 and HDAC2 (Fig. 2D). We then validated the targeting relationship between miR-495 and HDAC2 utilizing luciferase assay. It was evident that the luciferase activity was declined in the cells cotransfected with HDAC2 wild-type (WT) and miR-495 mimic, indicating that miR-495 could bind to the HDAC2 (Fig. 2E). Pearson’s correlation-coefficient analysis confirmed the negative linkage between HDAC2 and miR-495 (Fig. 2F).

Further, HDAC2 mRNA and protein expression were both reduced in PE tissues and primary trophoblast cells compared to that from healthy pregnant women (Fig. 2G, H; Supplementary Fig. S1B, 1C). The results also supported that HDAC2 expression was decreased upon miR-495 mimic (Fig. 2I, J; Supplementary Figs. S4A and 3B). These results indicated that HDAC2 was downregulated in PE tissues and could be targeted by miR-495.

### Overexpression of HDAC2 suppresses p53 expression by inducing deacetylation on p53

**Fig. 3:**
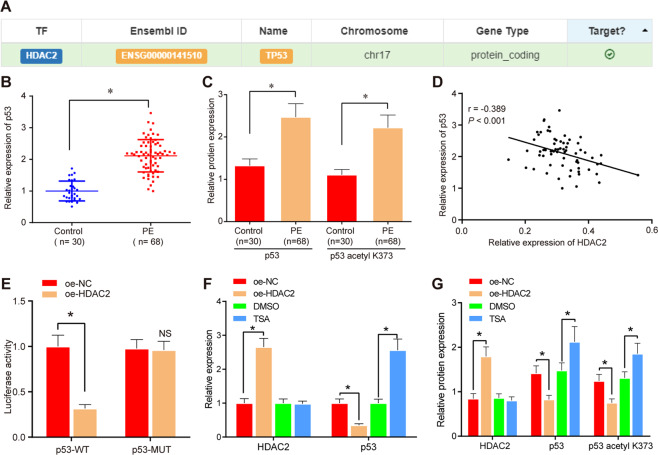
Overexpressed HDAC2 negatively regulates p53 expression in HTR8/SVneo cells. **A** hTFtarget prediction on the relationship between HDAC2 and p53 (TP53). **B** RT-qPCR to detect mRNA expression of p53 in umbilical cord tissues (control *n* = 30; PE *n* = 68). **C** Western blotting to analyze protein-expression level of p53 and p53 Ac in umbilical cord tissues (control *n* = 30, PE *n* = 68). **D** Analysis of the correlation between HDAC2 and p53. **E** Dual-luciferase reporter gene assay to verify the targeting relationship between HDAC2 and p53 in HTR8/SVneo cells. **F** RT-qPCR to analyze mRNA expression of p53 in HTR8/SVneo cells 48 h after transfection. **G**, Western blotting to detect the protein expression levels of p53 and p53 acetyl K373 in HTR8/SVneo cells 48 h after transfection. *p < 0.05 vs. oe-NC or DMSO. The quantitative data were presented as mean ± standard deviation. Difference between two groups was compared using paired *t-*test. Cell experiments were repeated 3 times.

To further identify the downstream mechanism of HDAC2, we adopted hTFtarget, which predicted that HDAC2 could target p53 (TP53) (Fig. 3A). We identified that the mRNA and protein expression of p53 and p53 acetyl K373 were both increased in PE tissues and primary trophoblast cells (Fig. 3B and C; Supplementary Fig. S1D, 1E). Pearson’s correlation coefficient showed a negative linkage between HDAC2 and p53 (Fig. 3D). Dual-luciferase reporter gene assay indicated that luciferase activity was reduced in response to p53-WT and oe-HDAC2 in HTR8/SVneo cells and primary trophoblast cells, indicating that HDAC2 could specifically bind to p53 (Fig. 3E; Supplementary Fig. S5A).

HTR8/SVneo cells and primary trophoblast cells were transfected with oe-NC and oe-HDAC2 or cultured with addition of dimethyl sulfoxide (DMSO) and inhibitor of HDAC Trichostatin A (TSA) small-molecule drugs, followed by expression determination. The results revealed that HDAC2 expression was increased, while expression of p53 and p53 acetyl K373 was decreased upon oe-HDAC2. Compared with DMSO-treated cells, the expression of HDAC2 was decreased, while the expression of p53 and p53 acetyl K373 was increased after TSA treatment (Fig. 3F, G; Supplementary Figs. S5B and 4C), which suggested that HDAC2 can deacetylate p53, leading to downregulation of p53 expression. These results showed that p53 was targeted by HDAC2 and overexpression of HDAC2 inhibited p53 expression by activating p53 deacetylation.

### p53 accelerates trophoblast cell growth by inducing upregulation of PUMA

**Fig. 4:**
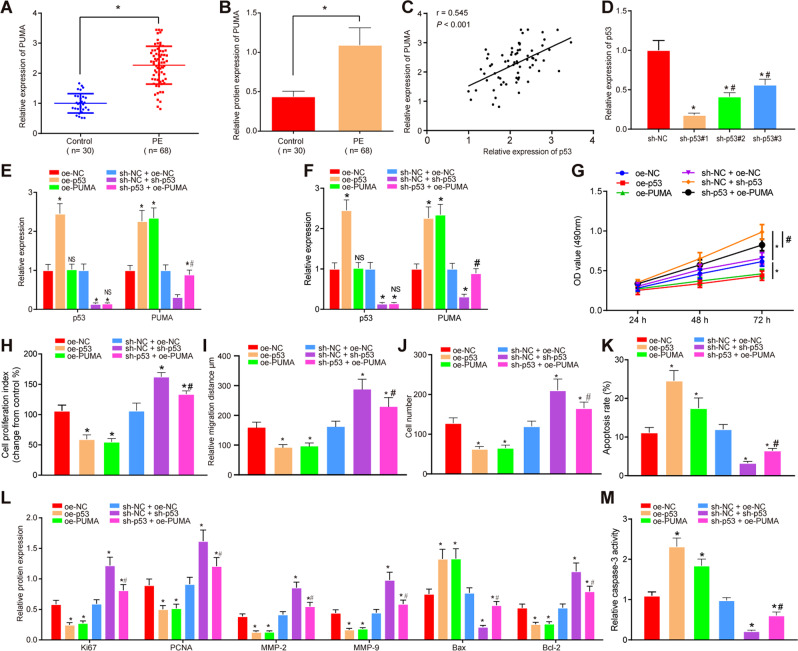
p53 upregulates PUMA expression to accelerate cell proliferation, invasion, and migration, but reduce apoptosis of HTR8/SVneo cells. **A** RT-qPCR detection on mRNA expression of PUMA (control *n* = 30, PE *n* = 68). **B** Western blot analysis on protein expression of PUMA (control *n* = 30, PE *n* = 68). **C** Correlation between p53 and PUMA expression analyzed by Pearson’s correlation coefficient. **D** RT-qPCR detection on knockdown efficiency of p53 in HTR8/SVneo cells. **E** mRNA expression of p53 and PUMA in each group of transfected HTR8/SVneo cells by RT-qPCR 48 h after transfection. **F** Protein expression of p53 and PUMA in each group of transfected HTR8/SVneo cells at 24^th^, 48^th^, and 72^nd^ hour after transfection by western blot. **G** HTR8/SVneo cell viability 48 h after transfection evaluated by MTT assay. **H** HTR8/SVneo cell proliferation 48 h after transfection evaluated by BrdU assay. **I** Migrating ability of HTR8/SVneo cells 24 h after transfection examined using scratch assay. **J** HTR8/SVneo cell invasion 24 h after transfection assessed using transwell assay. **K** HTR8/SVneo cell apoptosis in each group analyzed by flow cytometry 48 h after transfection. **L** Protein expression of Ki67, PCNA, MMP-2, MMP-9, Bax, and Bcl-2 in HTR8/SVneo cells 48 h after transfection studied by western blot. **M** Caspase-3 activity in HTR8/SVneo cells 48 h after transfection. *p < 0.05 vs. oe-NC or sh-NC + oe-NC; #*p* < 0.05 vs. sh-NC + sh-p53. The quantitative data were presented as mean ± standard deviation. Difference between two groups was analyzed using paired *t-*test. Data among multiple groups were processed using one-way ANOVA followed with Tukey’s post hoc test. Cell experiments were repeated 3 times.

PUMA is highly expressed in PE and can promote the occurrence of PE. PUMA is a downstream target gene of p53, and highly expressed p53 can promote PUMA expression [20–24]. Based on the above-related literature, we speculate that p53 promotes occurrence of PE by upregulating PUMA. To understand how p53/PUMA axis involved in PE, expression of PUMA in clinical umbilical cord tissues was analyzed. mRNA (Fig. 4A; Supplementary Fig. S1F) and protein (Fig. 4B; Fig. S1G) expression of PUMA in the PE tissues and primary trophoblast cells increased, suggesting that PUMA was upregulated in PE. Pearson’s correlation coefficient showed that p53 was positively correlated with PUMA expression (Fig. 4C). Next, HTR8/SVneo cells and primary trophoblast cells were transfected with oe-NC, oe-p53, oe-PUMA, short-hairpin RNA (sh)-NC, and sh-p53, followed by RT-qPCR to detect p53-knockdown efficiency, which showed that sh-p53#1 induced the most significantly downregulated p53 expression among the 3 shRNAs, thus the sh-p53#1 was selected for subsequent experiments (Fig. 4D; Supplementary Fig. S6A). Further, overexpression of p53 induced the expression of PUMA. In contrast, knockdown of p53 inhibited PUMA expression, while further overexpression of PUMA can reverse the effects of sh-p53 on PUMA expression (Fig. 4E, F; Supplementary Figs. S6B and 5C).

In addition, MTT assay (Fig. 4G; Supplementary Fig. S6D), BrdU assay (Fig. 4H; Supplementary Fig. S6E), scratch assay (Fig. 4I; Supplementary Fig. S6F), transwell assay (Fig. 4J; Supplementary Fig. S6G), and flow cytometry (Fig. 4K; Supplementary Fig. S6H) revealed that overexpression of p53 or upregulation of PUMA could inhibit cell viability, proliferation, migration, and invasion, but induce apoptosis of HTR8/SVneo cells and primary trophoblast cells compared with the oe-NC-treated cells, whereas opposite results were observed after p53 knockdown. However, sh-p53 and oe-PUMA in combination reversed the trends of p53 knockdown alone. Western blot demonstrated that after upregulation of p53 or PUMA, expression of Ki67, PCNA, MMP-2, MMP-9, and Bcl-2 was inhibited, yet Bax expression was promoted. However, depletion of p53 had opposite results, while further overexpression of PUMA reversed these trends (Fig. 4L; Supplementary Fig. S6I). The activity of caspase 3 was increased after upregulation of p53 or PUMA, while decreased after depletion of p53. However, sh-p53 and oe-PUMA in combination reversed the trends of p53 knockdown alone on caspase-3 activity (Fig. 4M; Supplementary Fig. S6J).

### HDAC2 promotes trophoblast cell growth by inactivating p53/PUMA pathway

**Fig. 5:**
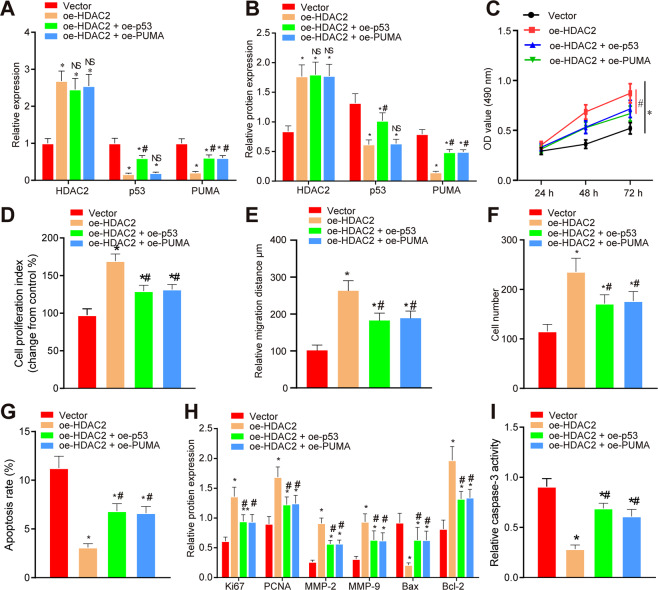
HDAC2 promotes proliferation, migration, and invasion, but inhibits apoptosis of HTR8/SVneo cells by regulating p53/PUMA axis. **A** RT-qPCR detection on the mRNA expression of HDAC2, p53, and PUMA 48 h after transfection. **B** Western blot analysis on protein expression of HDAC2, p53, and PUMA48 hours after transfection. **C** HTR8/SVneo cell viability at 24^th^, 48^th^, and 72^nd^ hour after transfection evaluated by MTT assay. **D** HTR8/SVneo cell proliferation 48 h after transfection evaluated by BrdU assay. **E** Migrating ability of HTR8/SVneo cells 24 h after transfection examined using scratch assay. **F** HTR8/SVneo cell invasion 24 h after transfection assessed using transwell assay. **G** HTR8/SVneo cell apoptosis in each group 48 h after transfection analyzed by flow cytometry. **H** Protein expression of Ki67, PCNA, MMP-2, MMP-9, Bax, and Bcl-2 in HTR8/SVneo cells 48 h after transfection studied by western blot. **I** Caspase-3 activity in HTR8/SVneo cells 48 h after transfection. *p < 0.05 vs. vector; #*p* < 0.05 vs. oe-HDAC2. The quantitative data were presented as mean ± standard deviation. Difference between two groups was compared using paired *t-*test. Data among multiple groups were analyzed using one-way ANOVA followed with Tukey’s post hoc test. Cell experiments were repeated 3 times.

HTR8/SVneo cells were overexpressed with HDAC2, p53, or PUMA. We observed elevated HDAC2 but reduced p53 and PUMA after oe-HDAC2 treatment alone or in combination with oe-p53 or oe-PUMA. Moreover, expression of p53 and PUMA was increased in the cells transfected with oe-HDAC2 or oe-p53 in the presence of oe-PUMA (Fig. 5A, B; Supplementary Figs. S7A and 6B). MTT assay (Fig. 5C; Supplementary Fig. S7C), BrdU (Fig. 5D; Supplementary Fig. S7D), scratch assay (Fig. 5E; Supplementary Figs. S3D and S7E), Transwell assay (Fig. 5F; Supplementary Figs. S3E and S7F), and flow cytometry (Fig. 5G; Supplementary Figs. S3F and S7G) pinpointed that the viability, proliferation, migration, and invasion of HTR8/SVneo cells and primary trophoblast cells were enhanced, while the cell apoptosis was decreased after all treatments compared with the vector. After cotransfection with oe-HDAC2 and oe-p53 or oe-PUMA, opposite findings were seen in the above-mentioned behaviors. Western blot showed that the expression of Ki67, PCNA, MMP-2, MMP-9, and Bcl-2 was increased, while the expression of Bax was decreased after all treatments than that in the vector control. Upon cotransfection with oe-HDAC2 and oe-p53 or oe-PUMA, the levels of Ki67, PCNA, MMP-2, MMP-9, and Bcl-2 protein were decreased, while the expression of Bax was increased (Fig. 5H; Supplementary Fig. S7H). Meanwhile, reduced caspase-3 activity was observed after all treatments relative to vector control, while caspase-3 activity was increased after cotransfection with oe-HDAC2 and oe-p53 or oe-PUMA (Fig. 5I; Supplementary Fig. S7I).

### miR-495 silencing inhibits p53/PUMA axis by upregulating HDAC2 to promote trophoblast cell growth

**Fig. 6:**
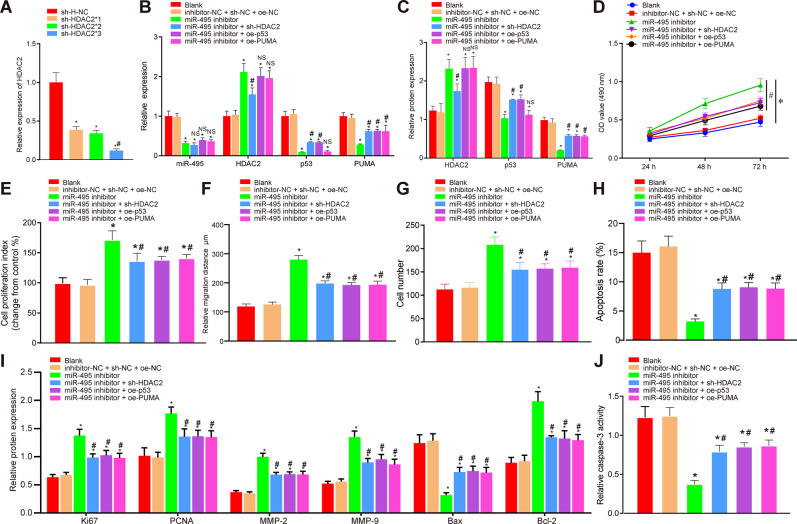
Downregulated miR-495 prevents PE by upregulating HDAC2 via inhibition of p53/PUMA axis in HTR8/SVneo cells. **A** Knockdown efficiency of HDAC2 analyzed by RT-qPCR. **B** miR-495, HDAC2, p53, and PUMA mRNA expression 48 h after transfection tested by RT-qPCR. **C** Western blot detection on protein expression of HDAC2, p53, and PUMA 48 h after transfection. **D** HTR8/SVneo cell viability evaluated by MTT assay. **E** HTR8/SVneo cell proliferation 48 h after transfection evaluated by BrdU assay. **F** Migrating ability of HTR8/SVneo cells 24 h after transfection examined using scratch assay. **G** HTR8/SVneo cell invasion 24 h after transfection assessed using transwell assay. **H** HTR8/SVneo cell apoptosis in each group 48 h after transfection analyzed by flow cytometry. **I** Protein expression of Ki67, PCNA, MMP-2, MMP-9, Bax, and Bcl-2 in HTR8/SVneo cells 48 h after transfection studied by western blot. **J** Caspase-3 activity in HTR8/SVneo cells 48 h after transfection. *p < 0.05 vs. blank or inhibitor NC + sh-NC + oe-NC; #*p* < 0.05 vs. miR-495 inhibitor. The quantitative data were presented as mean ± standard deviation. Difference between two groups was analyzed using paired *t-*test. Data among multiple groups were processed using one-way ANOVA followed with Tukey’s post hoc test. Cell experiments were repeated 3 times.

HTR8/SVneo cells were treated with miR-495 inhibitor alone or in combination with sh-HDAC2, oe-p53, or oe-PUMA. RT-qPCR validated the transfection efficiency of HDAC2. sh-HDAC2*3 showed the most significantly downregulated HDAC2 expression, and was selected for subsequent experiments (Fig. 6A; Supplementary Fig. S8A). We found that expression of miR-495, p53, and PUMA was decreased, while the expression of HDAC2 was increased in cells treated with miR-495 inhibitor alone or in combination with sh-HDAC2, oe-p53, or oe-PUMA. Moreover, the expression of HDAC2 was decreased, while the expression of p53 and PUMA was increased in cells cotransfected with miR-495 inhibitor and sh-HDAC2. The expression of p53 and PUMA was also increased in cells treated with miR-495 inhibitor and oe-p53. Increased PUMA was found in cells treated with miR-495 inhibitor and oe-PUMA (Fig. 6B, C; Supplementary Figs. S8B and 7C).

MTT assay (Fig. 6D; Supplementary Fig. S8D), BrdU (Fig. 6E; Supplementary Fig. S8E), scratch assay (Fig. 6F; Supplementary Fig. S8F), transwell assay (Fig. 6G; Supplementary Fig. S8G), and flow cytometry (Fig. 6H; Supplementary Fig. S8H) demonstrated that cell viability, proliferation, migration, and invasion of HTR8/SVneo and primary trophoblast cells were strengthened, but cell apoptosis was weakened in cells treated with miR-495 inhibitor alone or in combination with sh-HDAC2, oe-p53, or oe-PUMA. In comparison with the HTR8/SVneo and primary trophoblast cells treated with miR-495 inhibitor alone, the above-mentioned behaviors were reversed after the further treatment of sh-HDAC2, oe-p53, or oe-PUMA.

As demonstrated by western blot, the expression of Ki67, PCNA, MMP-2, MMP-9, and Bcl-2 was increased, while Bax expression was decreased after other treatments compared with the blank or cells cotreated with inhibitor NC, sh-NC, and oe-NC, while opposite trends observed were increased after treatment of sh-HDAC2, oe-p53, or oe-PUMA on the basis of miR-495 inhibitor (Fig. 6I; Supplementary Fig. S8I). Meanwhile, reduced caspase-3 activity was observed in cells treated with miR-495 inhibitor alone or in combination with sh-HDAC2, oe-p53, or oe-PUMA compared with the blank or NCs, while the opposite trend was observed in HTR8/SVneo and primary trophoblast cells treated with miR-495 inhibitor in combination with sh-HDAC2, oe-p53, or oe-PUMA compared with cells treated with miR-495 inhibitor alone (Fig. 6J; Supplementary Fig. S8J).

## Discussion

Trophoblast cells exert crucial action in the formation of placenta by invasion into myometrium to cause the onset of PE, within which the limited trophoblast proliferation, invasion, and migration were shown [25]. HTR8/SVneo trophoblast cells belong to a type of immortalized human extravillous trophoblast [26], which is used for the current studies. Even though PE has been studied extensively, the underlying mechanism remains elusive. Here, we discussed the role of miR-495/HDAC2/p53/PUMA crosstalk in the pathogenesis of PE. Our data indicated that miR-495 activated the p53/PUMA pathway to induce PE by limiting HDAC2.

Our work showed that miR-495 was highly expressed in placental tissues of patients with PE and induced trophoblast apoptosis, but restrained the cell proliferation, invasion, and migration. Consistent with our study, miR-495 is reported to be highly expressed in both mesenchymal stem cells and umbilical cord tissues of PE patients, which could promote the cell apoptosis [27]. Moreover, miR-495 expression shows a significant increase in the exosomes derived from mesenchymal stem cells in PE [13]. Moreover, we also found that miR-495 overexpression decreased the protein levels of Ki67, PCNA, MMP-2, and MMP-9, while increased the Bax expression in HTR8/SVneo cells and primary trophoblast cells. As common proliferation-related proteins Ki67 and PCNA, invasion- related proteins MMP-2 and MMP-9, as well as apoptosis-related protein Bcl-2, the increase in their levels is correlated with the increased proliferation and invasion of trophoblast cells in PE [28].

Our subsequent analysis elucidated that miR-495 could target HDAC2. As epigenetic regulators, HDACs are considered as therapeutic targets in cancer [29]. The HDAC2 expression in placental tissues of severe PE patients was decreased, which is considered as an inhibiting regulator to PE development [16]. Similar to our findings, there are other miRNAs that target HDAC2. For example, miR-145 functions through increasing the expression of HDAC2 [30]; miR-494-3p targets HDAC2 in lymphocytes in acute ischemic-stroke patients [31] and negatively regulates HDAC2 that can activate Nrf2/ARE signaling pathway by limiting Keap1, thereby accelerating osteoblast growth [32]. Our work revealed another miRNA, miR-495, which targets HDAC2 and plays important roles in PE development.

Moreover, we found that overexpression of HDAC2 suppressed p53 expression. A previous study reported that p53 protein was upregulated in human umbilical cord vein endothelial cells in PE, which was associated with the cellular proliferation in PE [33]. It is reported that p53 pathway was altered in villous trophoblast in PE, which was featured by apoptosis of villous trophoblast [25]. Consistent with our study, HDAC2 is considered as a regulator of p53-dependent gene expression to affect cell fate [34]. Specifically, HDAC2 could negatively regulate p53 protein expression, while depletion of HDAC2 induces cell death by activating p53 in lung disease [35]. As earlier clarified, in cells with ubiquitin-specific peptidase-4 overexpression, the accumulation of HDAC2 leads to the impairment of p53 acetyl k373 acetylation and p53 transcriptional activation during DNA damage [29]. The loss of HDAC1 and HDAC2 in mouse ureteral-bud (UB) cell line results in significant hyperacetylation of p53 on lysines 370, 379, and 383. These post-translational modifications are considered to improve the stability and transcriptional activity of p53 [36]. Our work demonstrated that miR-495 activated the p53/PUMA pathway by limiting HDAC2, which could lead to the progression of PE. PUMA, a pro-apoptotic marker, is highly expressed in PE patients [21]. It is also shown that hypoxia-induced factor-1 alpha (HIF1-alpha)-mediated PUMA accelerated PE progression [20]. PUMA expression can be upregulated by p53 to affect HeLa cell apoptosis [22], which is in line with our data that showed that upregulation of p53 enhanced PUMA expression in HTR8/SVneo cells and induced HTR8/SVneo cell apoptosis.

**Fig. 7:**
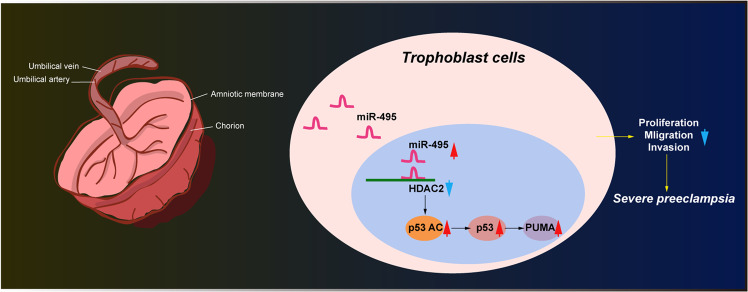
Schematic diagram of the mechanism by which miR-495 suppresses the expression of HDAC2 to activate p53/PUMA axis, inhibiting proliferation, migration and invasion, but promoting apoptosis of trophoblast cells, which may lead to the development of PE. The mechanism of how miR-495/HDAC2/p53/PUMA axis functions in the pathogenesis of PE.

In summary, our study demonstrated that miR-495 was highly expressed in placental tissues of patients with PE, but HDAC2 was downregulated. miR-495 activated p53/PUMA axis to inhibit the proliferation, invasion, and migration, but accelerate apoptosis of trophoblast cells by limiting HDAC2 in PE (Fig. 7). This study provides a novel miRNA-based therapeutic target against PE. Other mechanisms that are involved in the regulation of p53/PUMA-signaling pathways and PE development need further investigation.

## Materials and methods

### Ethics statement

All protocols involved in this study were ratified by the Ethics Committee of the First Hospital of China Medical University. Informed consents were obtained from patients, which was in the light of the *Declaration of Helsinki*.

### Bioinformatics analysis

The PE-related microarray GSE48424 containing 18 pairs of normal and PE samples was obtained from the Gene Expression Omnibus (GEO) database. Differential analysis was conducted using |logFoldChange| > 1 and *p-*value <0.05 as threshold. Target genes of miR-495 were predicted through the biological websites StarBase, RNAInter, and microT databases using different algorithms to match binding sites. The prediction results were analyzed using the Venn diagram online tool. Protein–protein interaction-network analysis was performed using STRING to study the hub protein for further analysis. Target proteins of hub genes were predicted through the hTFtarget website.

### Study subjects

Sixty-eight pregnant women who underwent cesarean section in the First Hospital of China Medical University from September 2015 to April 2017 were chosen as study subjects. Umbilical cord and placental tissues were collected immediately after full-term delivery, rinsed with phosphate buffer saline (PBS), and frozen at −80 °C for further use. Normal pregnant women (*n* = 30) who had no history of PE or any other complications, such as premature rupture of membranes, fetal malformations, maternal hypertension, or a history of kidney, heart disease, maternal infection, or smoking, were randomly selected as controls. The severe PE was evaluated as previously described [37, 38] (Table S1). The investigator was blinded to the group allocation during the experiment and/or when assessing the outcome.

### RT-qPCR

Total RNA extraction was implemented using the Trizol reagent (Invitrogen, Carlsbad, CA, USA). Complementary DNA (cDNA) was generated via reverse transcription using PrimeScript reverse transcription kit (Takara, Tokyo, Japan) and cDNA reverse transcription kit (K1622, Reanta Biotechnology, Beijing, China). The cDNA of miRNA containing PolyA tail was obtained using the Polya add-end test kit (B532451, Sangon, Shanghai, China). The quantitative PCR was implemented following the SYBR^®^ Premix Ex Taq^TM^ II kit (Takara) and the assay was performed using ABI 7500 fluorescence quantitative PCR instrument (Applied Biosystems, Foster City, CA, USA). Finally, 2^-ΔΔCt^ method was implemented for calculation of the relative expression with U6 and glyceraldehyde-3-phosphate dehydrogenase (GAPDH) as internal references. The experiments were repeated 3 times (Table S2).

### Western blot analysis

The umbilical cord tissues were homogenized (100 μL), placed in the reaction tube, lysed using 1 mL of protease-containing cell lysate for 30 min at 4 °C, shaken once every 10 min at 4 °C, and centrifuged at 6000 r/minute for 20 min. After the lipid layer was discarded, the supernatant was taken as protein extract. Protein concentration was measured utilizing a bicinchoninic acid kit (20201ES76, Yeasen Biotechnology, Shanghai, China). Proteins were loaded and separated by sodium dodecyl sulfate–polyacrylamide gel electrophoresis followed by transferring onto the polyvinylidene fluoride membrane. The membrane was blocked with 5% skim milk at room temperature for 1 h and incubated with primary rabbit anti-mouse antibodies (Abcam Inc., Cambridge, UK) to HDAC2 (ab32117, 1:2000), p53 (ab131442, 1:1000), p53 Ac (acetyl K373) (ab75754, 1:1000), PUMA (ab33906, 1:2000), Ki67 (ab92742, 1:5000), PCNA (ab152112, 1:1500), MMP-2 (ab37150, 1:2000), MMP-9 (ab73734, 1:2000), Bax (ab32503, 1:2000), Bcl-2 (ab59348, 1:1000), and GAPDH (ab8245, 1:5000) overnight at 4 °C. The next day, the membrane was reacted with horseradish peroxidase-labeled goat anti-rabbit immunoglobulin G (IgG) (ab6721, 1:5000) at room temperature for 1 h. The membrane was developed by ECL imager. Quantity One software was used to quantify the protein-expression level with GAPDH as an internal control.

### Isolation and evaluation of primary trophoblast cells

The placenta was stripped under aseptic conditions from 5 cases of PE patients and 5 cases of normal pregnant women and the placenta lobes were cut and washed with saline. pH 7.4, D-Hank’s containing 25 mmol/L HEPES served as digestive buffer, and added with a final concentration of 2.5 g/L, 300 U/mL DNaseI to prepare digestive fluid, followed by digestion at 37 °C. The supernatant was taken, centrifuged at 1000 g for 15 min, resuspended with L-DMEM, filtered in 200 mesh stainless steel, and resuspended after centrifugation. Percoll gradient-density separation was performed. Briefly, 4 mL of Percoll separation solution was placed layer by layer in the 50 mL centrifuge tube (1 × 7 density, each density/5 mL). The dilution scheme for preparation of Percoll gradients is listed in Table S3. The tube was slowly added with 5 mL of cell suspension, and centrifuged at 1200 g for 20 min. The liquid of 12.5–20.0 mL of the cell layer (trophoblast cells) was collected, and then centrifuged at 1000 g at room temperature for 15 min after dilution with D-Hank’s.

Trophoblast cells were evaluated. The extracted primary cells were resuspended with a DMEM/F12 containing 10% FBS. After the fibroblasts were removed using differential attachment method, a part of cells was added with 0.1 mL cell suspension and incubated with mouse anti-human CytoKeratin 7 antibody (ab218439, Abcam Inc.) and Annexin-V-fluoresceine isothiocyanate (FITC)-labeled sheep anti-mouse secondary antibody (ab6785, Abcam Inc.). The cell purity was determined with flow cytometry. Trophoblast cells with CytoKeratin-7 positive rate >90% were used for subsequent experiments.

### Cell culture

Human cell lines HTR8/SVneo (CRL-3271) were purchased from American Type Culture Collection (ATCC, USA) and HEK293T (SCSP-502) was purchased from the Cell Bank (Chinese Academy of Sciences, Shanghai, China). Cells were cultured in DMEM (12800017, Gibco BRL, Rockville) medium appended to 10% FBS (26140079, Gibco BRL) and 1% penicillin–streptomycin in an incubator (BB15, Thermo Fisher Scientific, San Francisco, CA) at 37 °C with a humidified atmosphere containing 5% CO_2_ and 95% O_2_. Cells were detached with 0.25% trypsin for 3 min, the digestion was terminated by the addition of DMEM medium appended to 10% FBS, and single-cell suspension was made via pipetting.

### Cell transfection

HTR8/Svneo cells and primary trophoblast cells were seeded in a 6-well plate 24 h before transfection. Upon 70% cell confluency, the cells were transfected following the instructions of Lipofectamine 2000 (11668019, Thermo Fisher Scientific). HTR8/SVneo cells and primary trophoblast cells were manipulated with miR-495 mimic/inhibitor, HDAC2-overexpression vector (oe-HDAC2), inhibitor of HDAC (TSA), DMSO, p53-overexpression vector (oe-p53), PUMA-overexpression vector (oe-PUMA), shRNA targeting p53 plasmid (sh-p53), empty vector, and the corresponding NC, individually or in combination. RNA-interference efficiency was analyzed using RT-qPCR by setting sh-p53#1, sh-p53#2, sh-p53#3, sh-HDAC2*1, sh-HDAC2*2, and sh-HDAC2*3. The shRNA with the highest silencing efficiency to p53 or HDAC2 was chosen for further experiments. All plasmids and vectors establishment, sequencing, lentivirus packaging, and testing were performed by Shanghai Genechem Technology Co., Ltd. (Shanghai, China). The detailed transfection sequences are shown in Table S4.

### Dual-luciferase reporter gene assay

WT and MUT 3′-untranslated region (3′-UTR) of HDAC2 was synthesized, followed by insertion into the pmiR-RB-REPORTTM vector (Guangzhou Ribobio Co., Ltd., Guangzhou, China). WT and MUT plasmids were cotransfected with miR-495 mimic or NC mimic into HEK293T cells, respectively. The luminescent signal was tested utilizing the dual-luciferase reporter analysis system (Promega, Madison, WI) with the relative light unit of Renilla luciferase detected by Renilla luciferase detection kit (YDJ2714, YuduoBio, Shanghai, China) normalized with firefly luciferase.

HTR8/SVneo cells and primary trophoblast cells were manipulated with a p53-Re promoter-driven luciferase construct and a Renilla luciferase construct (control) using Lipofectamine 2000. Cells were cultured for 24 h and incubated with 20 μm etoposide for 16 h. The luciferase activity was measured using the dual-luciferase system (Promega, San Luis, CA, USA) [25] to investigate whether HDAC2 could specifically bind to p53.

### MTT assay

Cultured cells were detached with 0.25% trypsin to prepare a single-cell suspension. Cells were seeded in a 96-well plate with a density of 5 × 10^6^−6 × 10^6^ cells per well in a volume of 0.2 mL per well and incubated in an incubator. The plates were removed at 24, 48, and 72 h after incubation, respectively, and replaced by the medium containing 10% MTT solution (5 g/L) (GD-Y1317, Gudo Biotechnology, Shanghai, China). After 4 h of incubation, each well was added with 100 μL of DMSO (D5879-100ml, Sigma-Aldrich, St. Louis, MO). The optical-density (OD) values were tested utilizing a microplate reader (BS-1101, Detie Lab, Nanjing, China) at 490 nm. The cell-viability curve was plotted against time points.

### BrdU assays

The cells to be tested were resuspended in a medium appended to 10% FBS and seeded in a 96-well flat microplate with a density of 1 × 10^4^ cells/well for BrdU cell-proliferation assay at the 48^th^ hour. BrdU was added to cells for incubation 24 h before the end of the test. BrdU was co-incubated with cells for 24 h and fixed with fixing solution at room temperature for 30 min. Finally, the plate was read at a dual wavelength of 450/550 nm utilizing a spectrophotometer microplate reader (TECAN, Infinite M200 Pro, Mannendorf, Switzerland). Three replicated wells were set for each experiment and experiments were repeated three times independently.

### Scratch assay

Transfected cells were incubated in an incubator at 37 °C in 5% CO_2_ and 95% O_2_ for 24 h. A line was gently created on the single-cell layer using a 10 μL pipette. Serum-free medium was added for another 24 h incubation. The cell migration at 0 and 24 h was evaluated under an inverted microscope and graphed with 3 randomly selected areas in each group. Experiments were repeated 3 times.

### Transwell assay

The Matrigel (40111ES08, Yeasen Biotechnology, Shanghai, China) diluted by serum-free DMEM (Matrigel: DMEM = 1:2) was coated on the Transwell apical chambers (3413, Unique Biotechnology, Beijing, China), which was solidified at 37 °C for 4–5 h. The transfected cells were diluted in 100 μL serum-free medium to prepare a cell suspension with a concentration of 1 × 10^6^ cells/mL and seeded in the apical chamber. The basolateral chamber was added with 500 μL DMEM medium containing 20% FBS. Each group was set up with 3 replicates. Transwell chambers were washed with PBS twice and cells were fixed with 5% glutaraldehyde and stained with 0.1% crystal violet for 5 min at 4 °C. The surface cells were removed with a cotton ball and assessed under an inverted fluorescence microscope (TE2000, Nikon, Tokyo, Japan). Five fields were randomly selected and graphed, and the number of cells passing through the chamber was recorded.

### Determination of caspase-3 activity

Based on the instructions, caspase-3 activity of HTR-8/SVneo cells was determined with a Caspase-3 assay kit (CASP3C-1KT, Sigma-Aldrich) after 48 h of transfection. The transfected cells were lysed with the lysis buffer from the kit, followed by centrifugation at 2000 g for 15 min. The supernatant of the lysed cells was collected and reacted with Ac-DEVE-pNA and reaction buffer. The optical density at 405 nm was determined as the caspase-3 activity.

### Flow cytometry

Apoptosis assay was implemented as an earlier-reported method [39] following flow cytometry (Bio-Rad ZE5, BIO-RAD, Hercules, CA) with the absorption wavelength of 488 nm and the excitation wavelength of 525 nm. The maximum absorption and emission wavelengths of the PI-DNA complex were 535 nm and 615 nm, respectively.

### Statistical analysis

Data were processed utilizing SPSS 21.0 (IBM, Armonk, NY, USA). The quantitative data were recorded by mean ± standard deviation. In the case of normal, distributed, and equal variance, unpaired *t*-test was chosen for data analysis between two groups, while one-way analysis of variance (ANOVA) for multiple-group comparisons, followed by Tukey’s post hoc test. Significant difference was pinpointed when *p* < 0.05.

## Supplementary information

Table S1

Table S2

Table S3

Table S4

Supplementary figure legends

Figure S1

Figure S2

Figure S3

Figure S4

Figure S5

Figure S6

Figure S7

Figure S8

## Data Availability

The datasets used and/or analyzed during the current study are available from the corresponding author on reasonable request.
