# Supplementary material for: MicroRNA-495 suppresses pre-eclampsia via activation of p53/PUMA axis
Source: Cell Death Discov. 2022 Mar 25;8:132. doi: 10.1038/s41420-022-00874-0 (PMC8956677; doi:10.1038/s41420-022-00874-0)
Supplement: Supplementary file 1 — Table S1 [file 41420_2022_874_MOESM1_ESM.docx]

**Table S1** Clinical characteristics of normal pregnancy and severe PE

|  | Control (n = 30) | PE (n = 68) | *p-*value |
| --- | --- | --- | --- |
| Childbearing age (year) | 27.2 ± 2.6 | 27.7 ± 3.1 | NS |
| Gestational age (weeks) | 38.1 ± 4.2 | 37.3 ± 4.5 | NS |
| SBP (mmHg) | 110.2 ± 16.7 | 167.7 ± 19.4 | < 0.05 |
| DBP (mmHg) | 75.3 ± 9.1 | 05.6 ± 6.5 | < 0.05 |
| Proteinuria (g/24h) | 0 | 4.7 ± 0.5 | < 0.05 |
| BUN(mmol/L) | 3.8 ± 0.5 | 3.9 ± 0.4 | NS |
| Fetal birth weight (g) | 3393.1 ± 301.7 | 2441.9 ± 254.3 | < 0.05 |

Notes: SBP, Systolic Blood Pressure; DBP, Diastolic Blood Pressure; BUN, Blood Urea Nitrogen; NS, No heterogeneity was found
