# Supplementary material for: MicroRNA-495 suppresses pre-eclampsia via activation of p53/PUMA axis
Source: Cell Death Discov. 2022 Mar 25;8:132. doi: 10.1038/s41420-022-00874-0 (PMC8956677; doi:10.1038/s41420-022-00874-0)
Supplement: Supplementary file 2 — Table S2 [file 41420_2022_874_MOESM2_ESM.docx]

**Table S2** Primer sequences for RT-qPCR

| Primers | Primer sequences |
| --- | --- |
| miR-495 | F: 5'-AAACAAACATGGTGCACTTCTT-3' |
|  | R: general primer |
| HDAC2 | F: 5'-TAAATCCAAGGACAACAGTGG-3' |
|  | R: 5'-GGTGAGACTGTCAAATTCAGG-3' |
| p53 | F: 5'-CTCCTCAGCATCTTATCCGAGTG-3' |
|  | R: 5'-GTGGTACAGTCAGAGCCAACC-3' |
| PUMA | F: 5'-CGGCGGAGACAAGAGGAGC-3' |
|  | R: 5'-CAGGGTGTCAGGAGGTGGGAG-3' |
| U6 | F: 5'-CTCGCTTCGGCAGCACA-3' |
|  | R: general primer |
| GAPDH | F: 5'-CTCTGATTTGGTCGTATTGGG-3' |
|  | R: 5'-TGGAAGATGGTGATGGGATT-3' |

Notes: miR-495, microRNA-495; HDAC2, Histone deacetylase 2; PUMA, p53 upregulated modulator of apoptosis; GAPDH, glyceraldehyde-3-phosphate dehydrogenase
