# Supplementary material for: MicroRNA-495 suppresses pre-eclampsia via activation of p53/PUMA axis
Source: Cell Death Discov. 2022 Mar 25;8:132. doi: 10.1038/s41420-022-00874-0 (PMC8956677; doi:10.1038/s41420-022-00874-0)
Supplement: Supplementary file 3 — Table S3 [file 41420_2022_874_MOESM3_ESM.docx]

**Table S3** Dilution scheme for preparation of Percoll gradients

| 90% Percoll | D-Hank’s | Density | Final concentration |
| --- | --- | --- | --- |
| (mL) | (mL) |  | (%) |
| 14.4 | 5.6 | 1.085 | 65 |
| 12.2 | 7.8 | 1.072 | 55 |
| 10 | 10 | 1.059 | 45 |
| 7.8 | 12.2 | 1.046 | 35 |
| 5.6 | 14.4 | 1.033 | 25 |
| 3.3 | 16.7 | 1.02 | 15 |
| 1.1 | 18.9 | 1.007 | 5 |
