# Supplementary material for: MicroRNA-495 suppresses pre-eclampsia via activation of p53/PUMA axis
Source: Cell Death Discov. 2022 Mar 25;8:132. doi: 10.1038/s41420-022-00874-0 (PMC8956677; doi:10.1038/s41420-022-00874-0)
Supplement: Supplementary file 4 — Table S4 [file 41420_2022_874_MOESM4_ESM.docx]

**Table S4** Transfection sequence for plasmids

| Plasmids | Transfection sequence |
| --- | --- |
| miR-495 mimic | 5′-UGUGACGAAACAAACAUGGUGCACU-3ʹ |
| miR-NC | 5′-CAGUACUUUUGUGUAGUACAA-3′ |
| miR-495 inhibitor | 5′-GCCGAAUUCUGGCUGCUAUGAUCUGAACU-3ʹ |
| inhibitor NC | 5′-CAGUACUUUUGUGUAGUACAA-3′ |
| sh-p53#1 | 5ʹ-CCGGCACCATCCACTACAACTACATCTCGAGATGTAGTTGTAGTGGATGGTGTTTTTG-3ʹ |
| sh-p53#2 | 5′-CCGGTCAGACCTATGGAAACTACTTCTCGAGAAGTAGTTTCCATAGGTCTGATTTTT-3′ |
| sh-p53#3 | 5′-CCGGGTCCAGATGAAGCTCCCAGAACTCGAGTTCTGGGAGCTTCATCTGGACTTTTTG-3′ |
| sh-HDAC2*1 | 5’-CCGGGACGGTATCATTCCATAAATACTCGAGTATTTATGGAATGATACCGTC-TTTTTG-3ʹ |
| sh-HDAC2*2 | 5’-CCGGCAGTCAAAGGTCATGCTAAATCTCGAGATTTAGCATGACCTTTGACTGTTTTTTG-3ʹ |
| sh-HDAC2*3 | 5’-CCGGGCCTATTATCTCAAAGGTGATCTCGAGATCACCTTTGAGATAATAGGCTTTTT-3ʹ |
| sh-NC | 5ʹ-CCTAAGGTTAAGTCGCCCTCG-3ʹ |

Notes: miR-495, microRNA-495; HDAC2, Histone deacetylase 2; NC, negative control
