# Supplementary material for: MicroRNA-495 suppresses pre-eclampsia via activation of p53/PUMA axis
Source: Cell Death Discov. 2022 Mar 25;8:132. doi: 10.1038/s41420-022-00874-0 (PMC8956677; doi:10.1038/s41420-022-00874-0)
Supplement: Supplementary file 5 — Supplementary figure legends [file 41420_2022_874_MOESM5_ESM.docx]

Supplementary figure legends

**Figure S1. The expression of miR-495, HDAC2, p53, and PUMA in the primary trophoblast cells from PE patient normal pregnant women.**

A, RT-qPCR detection of expression of miR-495 in primary trophoblast cells from PE patients. B, RT-qPCR detection of HDAC2 mRNA expression in primary trophoblast cells. C, Western blot analysis of HDAC2 protein expression in primary trophoblast cells. D: RT-qPCR detection of mRNA expression of p53 in primary trophoblast cells. E, Western blot analysis of protein expression of p53 in primary trophoblast cells. F, RT-qPCR detection of PUMA mRNA expression in primary trophoblast cells. G, Western blot analysis of PUMA protein expression in primary trophoblast cells. **p* < 0.05 *vs.* control group (control n = 5; PE n = 5).

**Figure S2. miR-495 inhibits cell proliferation, invasion and migration, but promotes apoptosis of primary trophoblast cells.**

A, Expression of miR-495 in primary trophoblast cells via RT-qPCR. B, Cell viability of primary trophoblast cells evaluated by MTT assay. C, Primary trophoblast cell proliferation evaluated by BrdU assay. D, Migrating ability of primary trophoblast cells examined using scratch assay. E, Primary trophoblast cell invasion assessed using transwell assay (200 ×). F, Primary trophoblast cell apoptosis analyzed by flow cytometry. G, Protein expression of Ki67 and PCNA, mMMP-2 and MMP-9 and Bax and Bcl-2 in primary trophoblast cells studied by western blot. H, Caspase 3 activity in primary trophoblast cells. **p <* 0.05 control or NC-mimic. The quantitative data were presented as mean ± standard deviation. Difference between two groups was compared using paired *t-*test. Cell experiments were repeated 3 times.

**Figure S3.** A, Representative image of Figure 1E. B, Representative image of Figure 1F. C, Representative image of Figure 1G. D, Representative image of Figure 5E. E, Representative image of Figure 5E. F, Representative image of Figure 5E.

**Figure S4. miR-495 negatively targets HDAC2 expression in primary trophoblast cells.**

A, RT-qPCR detection on HDAC2 expression in primary trophoblast cells after transfection. B, Western blot analysis on HDAC2 expression in primary trophoblast cells after transfection. **p <* 0.05 control or NC-mimic. The quantitative data were presented as mean ± standard deviation. Difference between two groups was analyzed by paired *t-*test. Cell experiments were repeated 3 times.

**Figure S5. HDAC2 negatively regulates p53 expression in primary trophoblast cells.**

A, Dual-luciferase reporter gene assay to verify the targeting relationship between HDAC2 and p53 in primary trophoblast cells. B. RT-qPCR to analyze mRNA expression of p53 in primary trophoblast cells; C, Western blot to detect the protein expression levels of p53 and p53 acetyl K373 in primary trophoblast cells. **p <* 0.05 *vs.* oe-NC or DMSO. The quantitative data were presented as mean ± standard deviation. Difference between two groups was compared using paired *t-*test. Cell experiments were repeated 3 times.

**Figure S6. p53 upregulates PUMA expression to participate in PE.**

A, RT-qPCR detection on knockdown efficiency of p53 in primary trophoblast cells. B, mRNA expression of p53 and PUMA in each group of transfected primary trophoblast cells by RT-qPCR. C, Protein expression of p53 and PUMA in each group of transfected primary trophoblast cells by western blot. D, Primary trophoblast cell viability evaluated by MTT assay. E, Primary trophoblast cell proliferation evaluated by BrdU assay. F, Migrating ability of primary trophoblast cells examined using scratch assay. G, Primary trophoblast cell invasion assessed using transwell assay. H, Primary trophoblast cell apoptosis in each group analyzed by flow cytometry. I, Protein expression of Ki67 and PCNA, MMP-2 and MMP-9, Bax and Bcl-2 in primary trophoblast cells studied by western blot. J, Caspase 3 activity in primary trophoblast cells. **p <* 0.05 *vs.* oe-NC or sh-NC + oe-NC; #*p* < 0.05 *vs.* sh-NC + sh-p53. The quantitative data were presented as mean ± standard deviation. Difference between two groups was analyzed using paired *t-*test. Data among multiple groups were processed using one-way ANOVA followed with Tukey’s post hoc test. Cell experiments were repeated 3 times.

**Figure S7. HDAC2 promotes proliferation, migration and invasion, but inhibits apoptosis of primary trophoblast cells by inhibiting p53/PUMA axis.**

A, RT-qPCR detection on the mRNA expression of HDAC2, p53 and PUMA. B, Western blot analysis on protein expression of HDAC2, p53 and PUMA. C, Primary trophoblast cell viability evaluated by MTT assay. D, Primary trophoblast cell proliferation evaluated by BrdU assay. E, Migrating ability of primary trophoblast cells examined using scratch assay. F, Primary trophoblast cell invasion assessed using transwell assay (200 ×). G, Primary trophoblast cell apoptosis in each group analyzed by flow cytometry. H, Protein expression of Ki67, PCNA, MMP-2, MMP-9, Bax and Bcl-2 in primary trophoblast cells studied by western blot. I, Caspase 3 activity in primary trophoblast cells. **p <* 0.05 *vs.* vector; #*p* < 0.05 *vs.* oe-HDAC2. The quantitative data were presented as mean ± standard deviation. Difference between two groups was compared using paired *t-*test. Data among multiple groups were analyzed using one-way ANOVA followed with Tukey’s post hoc test. Cell experiments were repeated 3 times.

**Figure S8. Downregulated miR-495 prevents PE by upregulating HDAC2 *via* inhibition of p53/PUMA axis.**

A, Knockdown efficiency of HDAC2 analyzed by RT-qPCR. B, miR-495, HDAC2, p53 and PUMA mRNA expression tested by RT-qPCR. C, Western blot detection on protein expression of HDAC2, p53 and PUMA. D, Primary trophoblast cell viability evaluated by MTT assay. E, Primary trophoblast cell proliferation evaluated by BrdU assay. F, Migrating ability of primary trophoblast cells examined using scratch assay. G, Primary trophoblast cell invasion assessed using transwell assay. H, Primary trophoblast cell apoptosis in each group analyzed by flow cytometry. I, Protein expression of Ki67, PCNA, MMP-2, MMP-9, Bax and Bcl-2 in primary trophoblast cells studied by western blot. J, Caspase 3 activity in primary trophoblast cells. **p <* 0.05 *vs.* blank or inhibitor NC + sh-NC + oe-NC; #*p* < 0.05 *vs.* miR-495 inhibitor. The quantitative data were presented as mean ± standard deviation. Difference between two groups was analyzed using paired *t-*test. Data among multiple groups were processed using one-way ANOVA followed with Tukey’s post hoc test. Cell experiments were repeated 3 times.
