# Supplementary figures and images for: MicroRNA-495 suppresses pre-eclampsia via activation of p53/PUMA axis
Source: Cell Death Discov. 2022 Mar 25;8:132. doi: 10.1038/s41420-022-00874-0 (PMC8956677; doi:10.1038/s41420-022-00874-0)

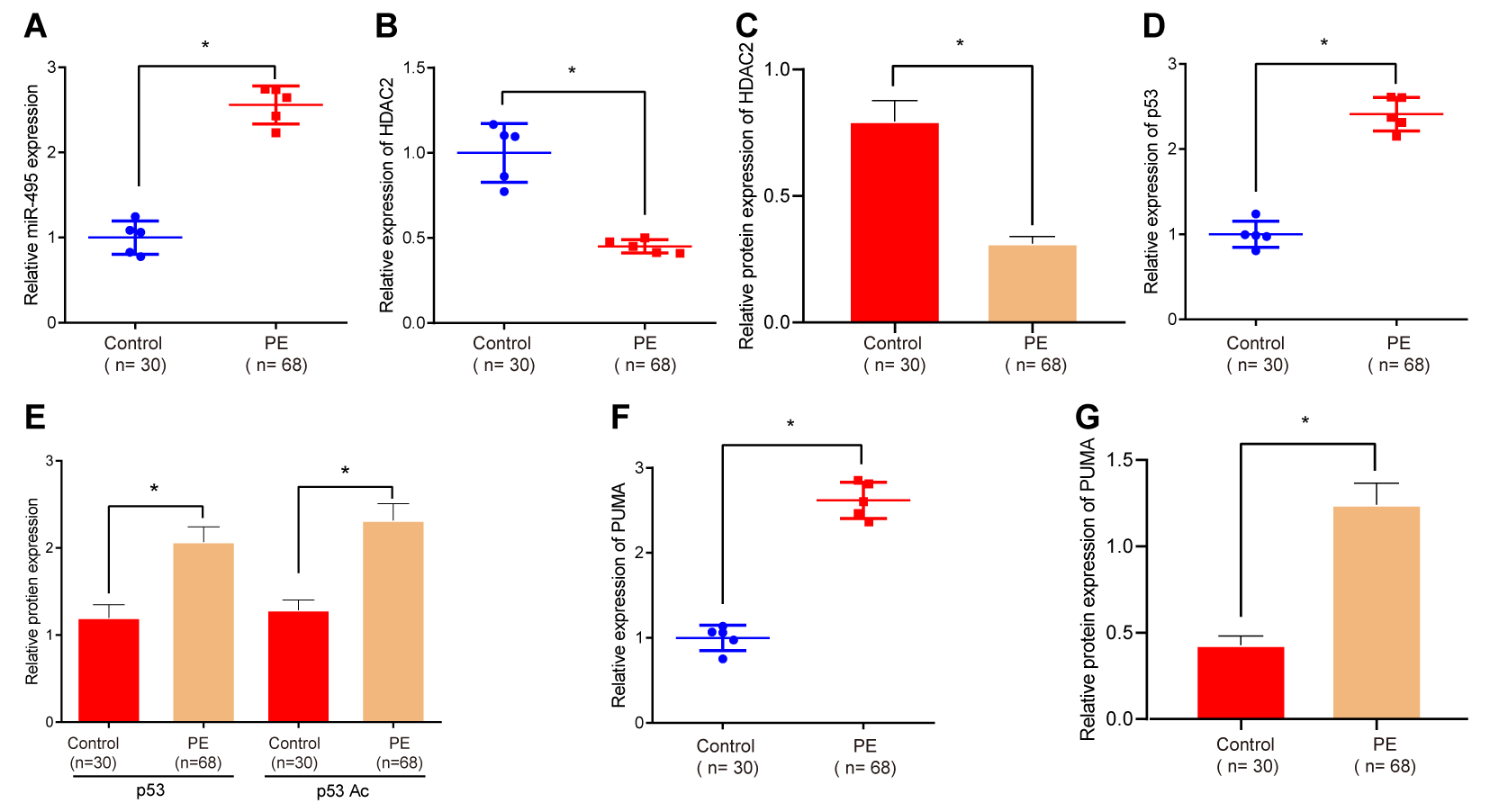

Supplement: Supplementary file 6 — Figure S1 [file 41420_2022_874_MOESM6_ESM.tif]

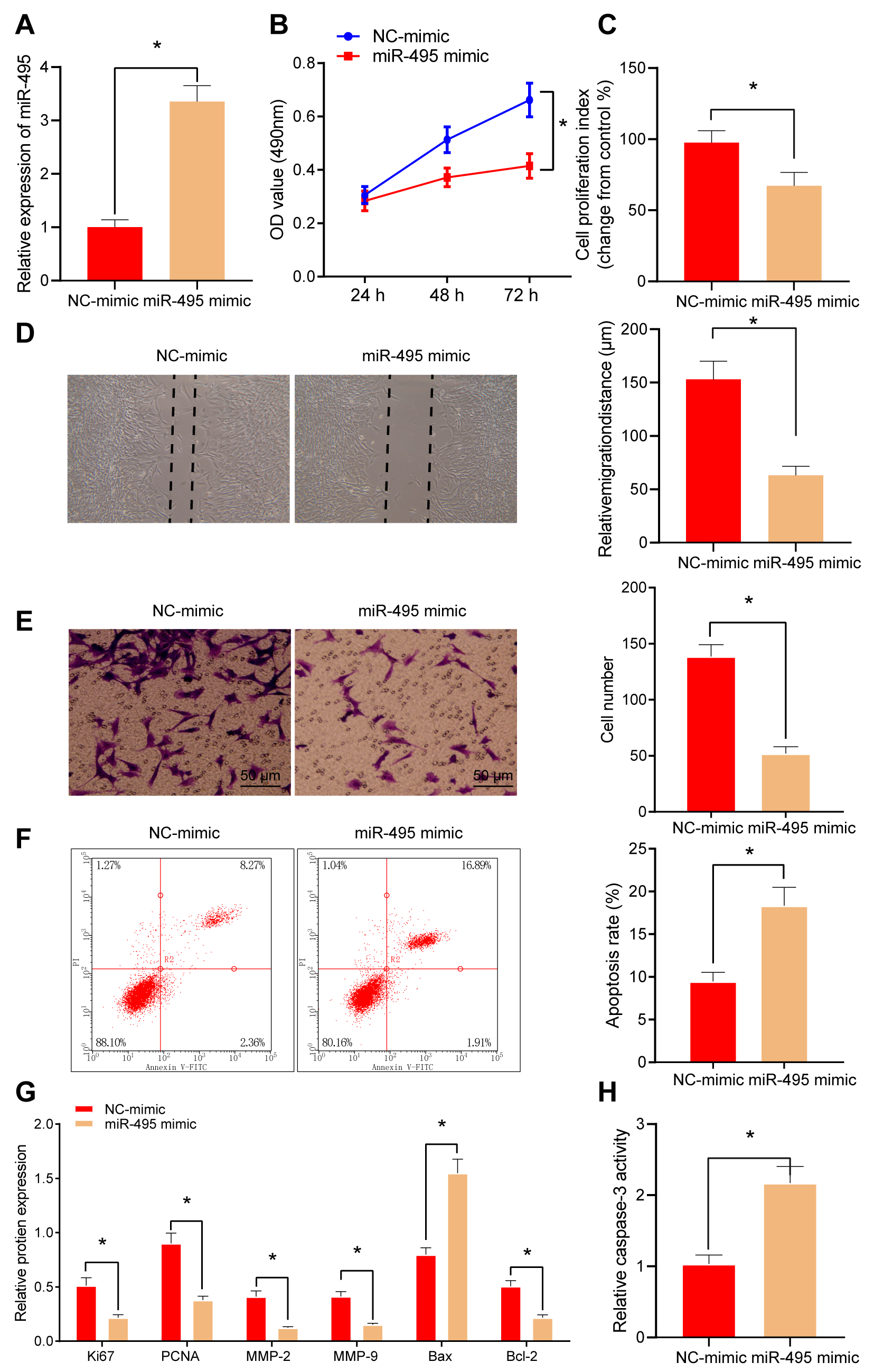

Supplement: Supplementary file 7 — Figure S2 [file 41420_2022_874_MOESM7_ESM.tif]

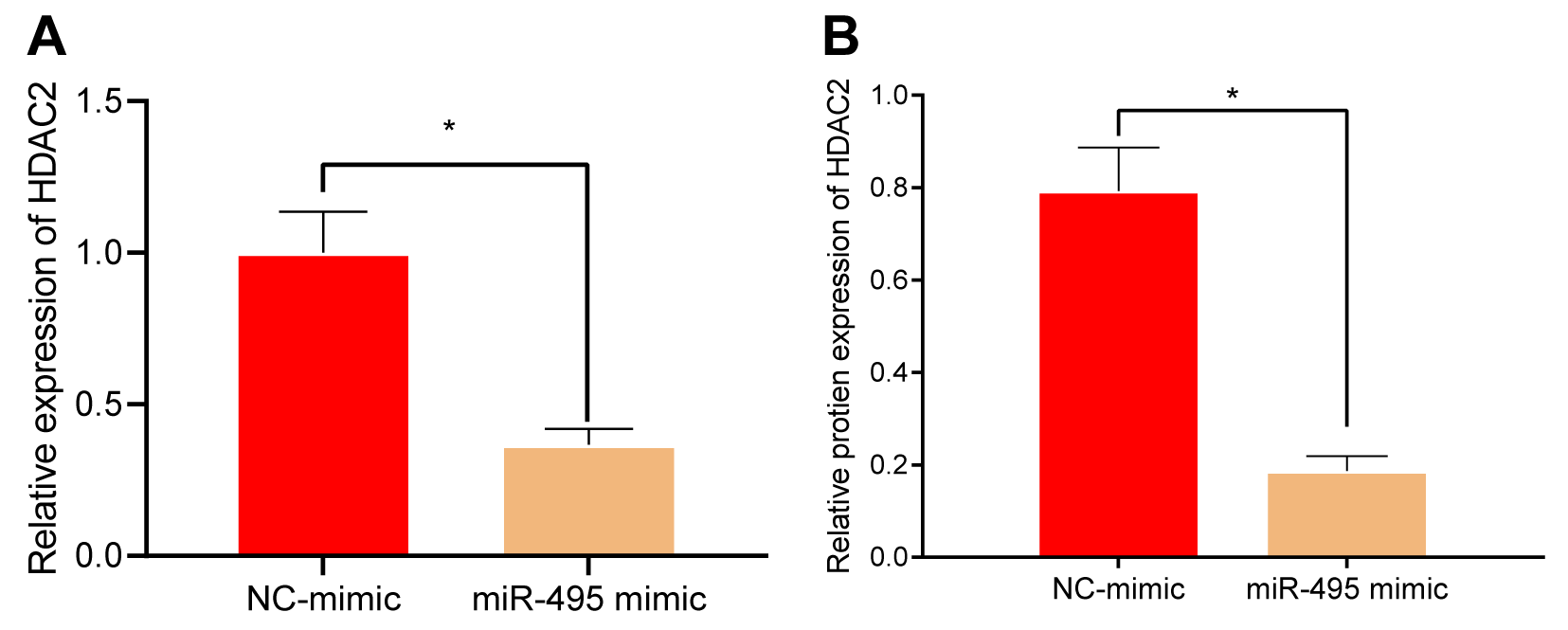

Supplement: Supplementary file 8 — Figure S3 [file 41420_2022_874_MOESM8_ESM.tif]

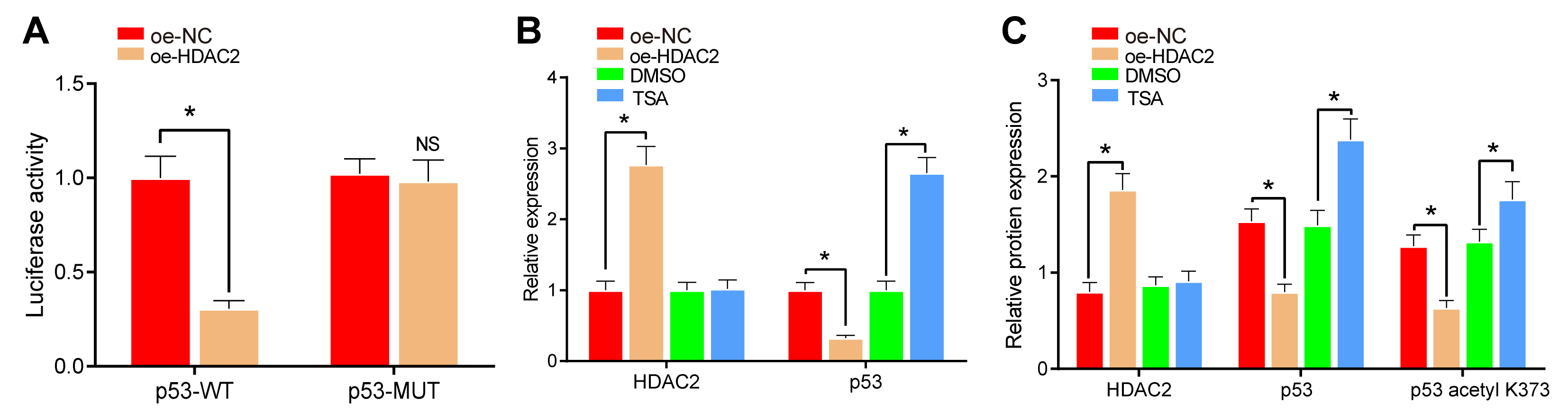

Supplement: Supplementary file 9 — Figure S4 [file 41420_2022_874_MOESM9_ESM.tif]

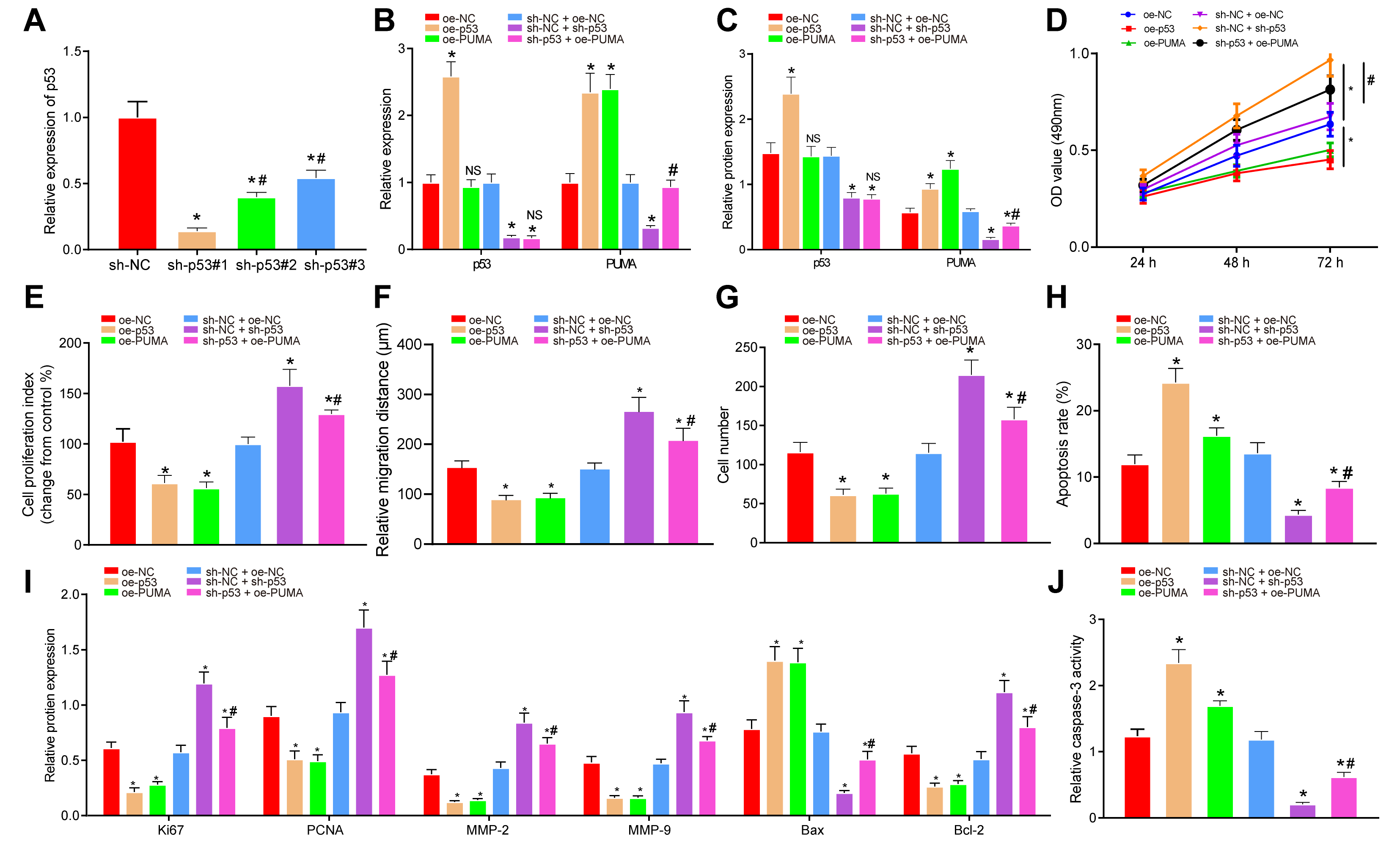

Supplement: Supplementary file 10 — Figure S5 [file 41420_2022_874_MOESM10_ESM.tif]

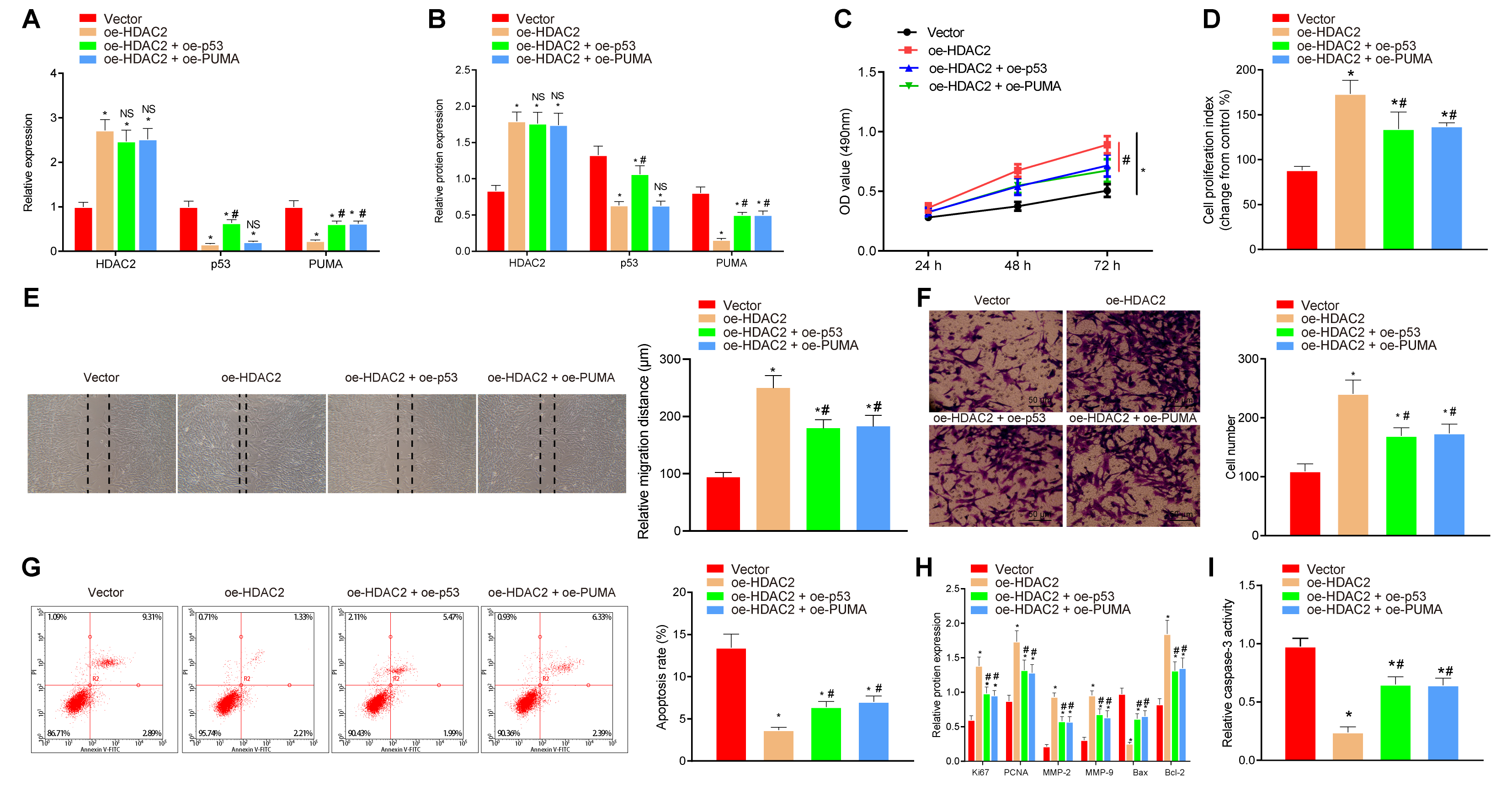

Supplement: Supplementary file 11 — Figure S6 [file 41420_2022_874_MOESM11_ESM.tif]

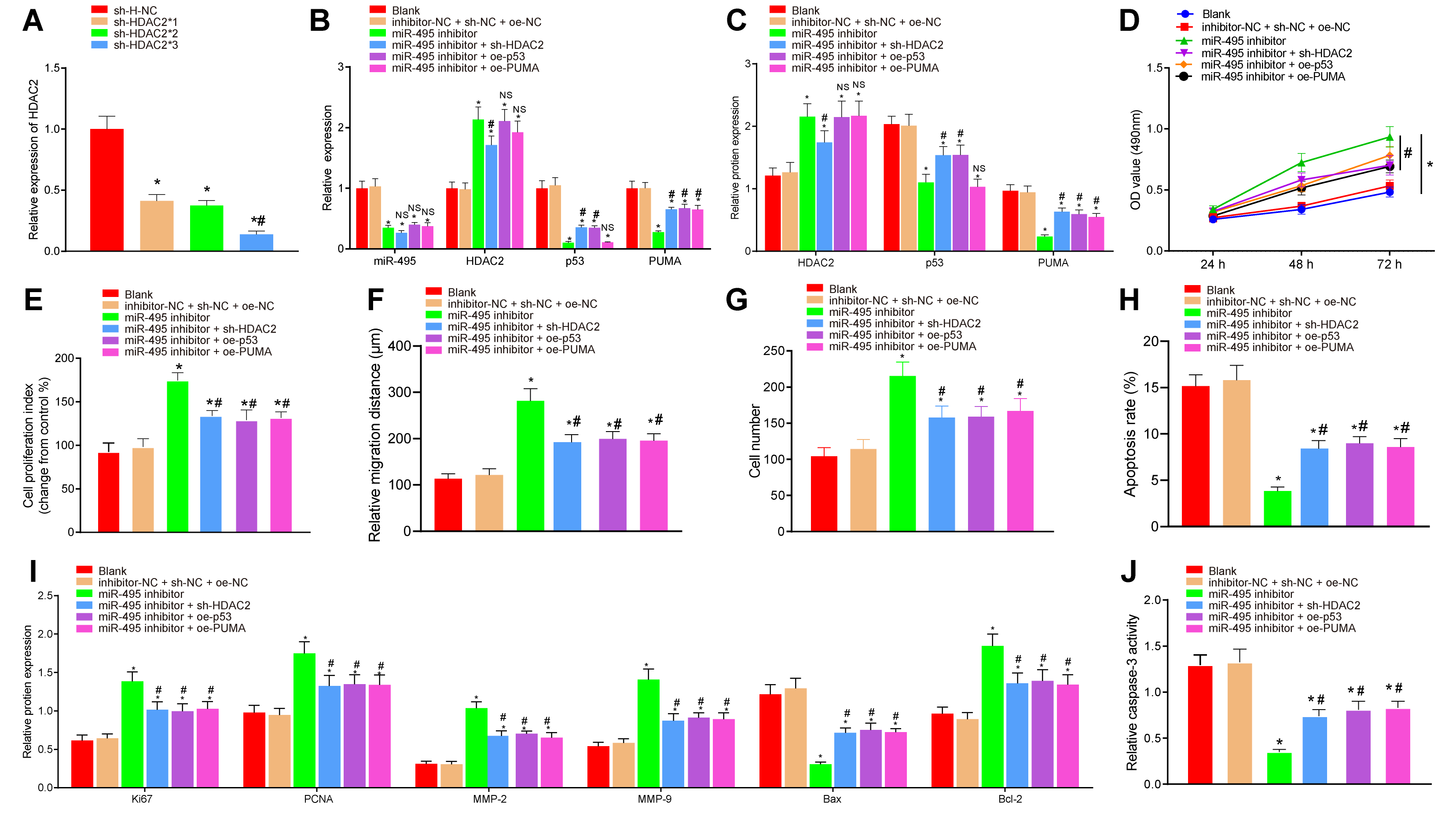

Supplement: Supplementary file 12 — Figure S7 [file 41420_2022_874_MOESM12_ESM.tif]

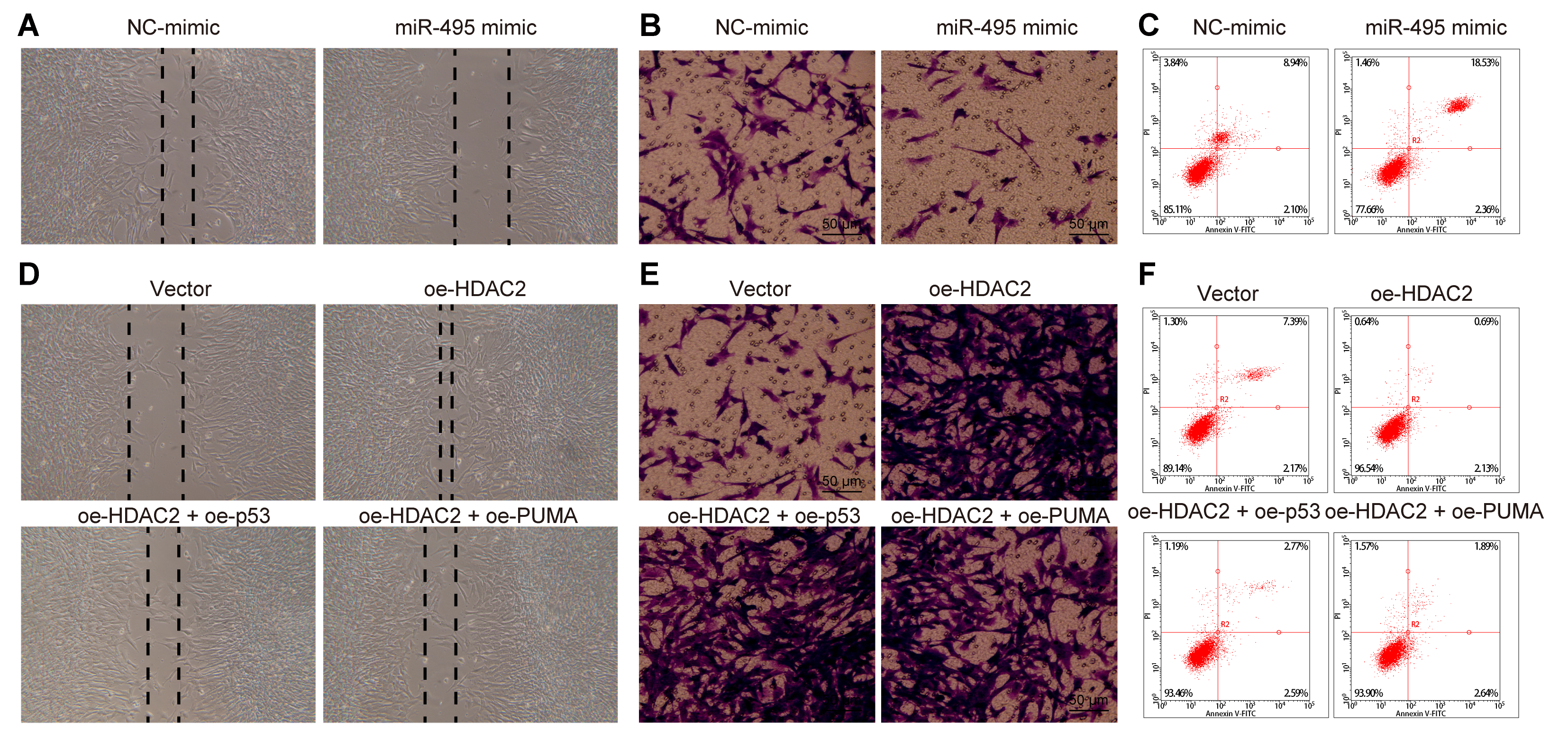

Supplement: Supplementary file 13 — Figure S8 [file 41420_2022_874_MOESM13_ESM.tif]
